# Dual Sensing Performance of 1,2-Squaraine for the Colorimetric Detection of Fe^3+^ and Hg^2+^ Ions

**DOI:** 10.3390/ma11101998

**Published:** 2018-10-16

**Authors:** Xiaoqian Liu, Na Li, Min-Min Xu, Chunhui Jiang, Jianhao Wang, Guoqiang Song, Yong Wang

**Affiliations:** 1 School of Pharmaceutical Engineering and Life Science, Changzhou University, Changzhou 213164, China; 16109214@smail.cczu.edu.cn (N.L.); minuswan@cczu.edu.cn (J.W.); 2 College of Chemistry, Chemical Engineering and Materials Science, Soochow University, Suzhou 215123, China; xumm@suda.edu.cn; 3 School of Environmental and Chemical Engineering, Jiangsu University of Science and Technology, 2 Mengxi Road, Zhenjiang 212003, China; chemjiang@just.edu.cn

**Keywords:** 1,2-squaraine, colorimetric sensor material, iron, mercury, molecular modelling, DFT calculation

## Abstract

A simple 1,2-squaraine based chemosensor material (SQ) has been reported to show dual sensing performance for colorimetric detection of Fe^3+^ and Hg^2+^ ions. Compared to common instrumental analysis, this method could provide fast and direct detection though colorimetric changes by the naked eye. The sensor has shown excellent selectivity over the other metal ions by tuning different solvent environments. The detection limit for Fe^3+^ could reach to 0.538 μM, which was lower than that in the environmental agency guideline (U.S. Environmental Protection Agency, U.S. EPA) in drinking water. And for Hg^2+^ detection, the limit was calculated as 1.689 μM in our case. A 1:1 binding mode between SQ–Fe^3+^ and SQ–Hg^2+^ ion were evidenced by Job’s plot measurement and IR analysis. The proposed different binding mechanisms were also supported by Density Function Theory (DFT) calculation. All these findings provide a unique material and a simple, facile, and low cost colorimetric method for dual metal ions analysis and have shown preliminary analytical applications in industrial water sample analysis.

## 1. Introduction

Development of selective and sensitive chemosensor materials for metal cation ions has attracted considerable attention owing to their key role and potential applications in chemistry, materials, and the environment [1,2,3,4,5,6,7,8,9,10,11]. Among heavy metals, iron is one of the essential elements playing an important function in a wide range of biological process, such as cellular metabolism, oxygen carrying, and regulation of enzyme reactions [12,13,14,15,16]. Meanwhile, mercury is considered a toxic element that has harmful effects on the human health and the environment. Accumulation of mercury in the body will cause severe health problem, especially damage of the central nervous system [17,18,19,20]. Therefore, a convenient and rapid method for the analysis of Fe^3+^ and Hg^2+^ is highly in demand. Current analytical techniques such as atomic absorption spectroscopy, inductively coupled plasma mass spectroscopy, and electrochemical analysis require sophisticated instruments and complex sample preparation procedures, which limit their wide applications [21,22,23,24,25]. It is imperative to develop simple material and effective methods for the detection of these ions. The use of a chemosensor material provides unique advantages in a view of sensitivity and response time. One strategy for the design of such molecules involves introducing an appropriate binding ligand to the chromophore in which optical responses are affected by the complexation process [26,27]. A serious of chromophore based materials has been developed, including squaraines, fluorescein, rhodamine, quinolones, porphyrins, coumarin, etc. [28,29,30,31,32,33,34]. However, design of a binding group which will be highly selective to each ion individually is not an easy proposition considered many ions share similar optical properties. Recently, another strategy for applying one single chemosensor material in simultaneous colorimetric detection of multiple metal ions has attracted great interests. The detection can be straightforward as long as the analysis shows differential responses to the metal ions. The strategy allows minimizing the multistep organic synthesis and accelerating the discovery process. A few examples have been reported to achieve multiple ion detections [35,36,37,38,39,40]. However, materials for dual detection of Fe^3+^ and Hg^2+^ are relatively rare. In this context, the design and synthesis of simple, facile, low cost colorimetric material for selectively recognition of Fe^3+^ and Hg^2+^ remains a challenge.

Squaraines are a class of versatile organic dyes which exhibit unique optical properties, such as intense absorption and efficient fluorescence emission in the visible to near infrared region [41,42]. They can be versatile by linking different electron donors based on heterocyclic structure such as aniline, indole group to the squaric core which acts as an electron acceptor [43,44,45,46,47]. Squaraines are suitable for use in chemosensors because the optical properties can be tuned by external factors such as change in polarity and pH of solvent, temperature or the addition of additives. We are gratifying to present 3,4-bis((Z)-(3-butylbenzo[d]thiazol-2(3H)-ylidene)methyl) cyclobut-3-ene-1,2-dione (SQ) to perform dual colorimetric sensing of Fe^3+^ and Hg^2+^, spontaneously, by tuning the solution environment. The sensor was sensitive in diverse solvent environments. It gave a selective color change from orange to cream yellow for addition of Fe^3+^ in acetic acid with a limit of 0.538 μM which was much lower than the limit in the U.S. environmental protection agency guideline (5.37 μM), while presenting a color change from orange to grain yellow for Hg^2+^ detection in the high concentrations of anionic surfactant sodium dodecyl sulfonate (SDS) solution with a detection limit of 1.689 μM. The binding constants and properties of 1,2-squanraine towards these two metal ions were further investigated by Job’s plot measurements, IR spectrums, and Election Spray Ionization-Mass spectra (ESI-MS), respectively. Other excess metal ions as interferences in the system show both negligible effects on the 1,2-squaraine towards Fe^3+^ and Hg^2+^ detections. Two different complexation patterns were then proposed. The two adjacent oxygen atoms at the electron deficient cyclobutene ring provided extra electron pairs with Fe^3+^ while the complexation towards Hg^2+^ differed another way. Further Density Function Theory (DFT) calculations have supported the hypothesis. Finally, the SQ material has proven to be successful in real sample applications.

## 2. Materials and Methods 

### 2.1. Chemicals and Materials

Unless stated, all the chemicals used were purchased from commercial sources without purification. ^1^H NMR (400 MHz) and ^13^C NMR (400 MHz) spectra were recorded on a Bruker AV-400 spectrometer (Bruker, Beijing, China, Tetramethylsilane as internal standard). Mass spectrometry analysis was performed on a Q Exactive mass spectrometer (Thermo Fisher Scientific, Shanghai, China). Absorption spectra were measured on Molecular Device Spectrometer 5 (Molecular Devices Corporation, San Jose, CA, USA). Infrared spectra were performed on a Digilab FTS-3000 FT-IR spectrophotometer (Digilab, Hopkinton, MA, USA).

### 2.2. General Procedures for UV-Vis Experiments

A stock solution of SQ was prepared 10 mM in dimethyl sulfoxide, DMSO. Further dilutions were made to prepare 100 μM of SQ by adding different solutions. Heavy metal ions stock solutions were prepared 10 mM in distilled water and diluted further accordingly. In UV-Vis experiments, 2 μL of 10 mM SQ solution and 2 μL of 10 mM heavy metal ions were extracted from stock solution and diluted with 196 μL of solvent to make a total volume of 200 μL. In this case, the concentration for SQ was fixed at 100 μM (100 time dilutions from the stock solution). After mixing for 1 min, the absorption measurements were made in 96 well plates on Molecular Device Spectrometer 5 (Molecular Devices Corporation, San Jose, CA, USA) at the wavelength range of 350 nm to 750 nm. 

### 2.3. Job’s Plot Measurements

The stock solutions of sensor SQ (10 mM) in DMSO and FeCl_3_ (10 mM) in distilled water were prepared, respectively. 0.2 μL, 0.4 μL, 0.6 μL, 0.8 μL, 1.0 μL, 1.2 μL, 1.4 μL, 1.6 μL, 1.8 μL of the sensor SQ (10 mM) solution were taken and transferred to the vials. 1.8 μL, 1.6 μL, 1.4 μL, 1.2 μL, 1.0 μL, 0.8 μL, 0.6 μL, 0.4 μL, 0.2 μL of FeCl3 (10 mM) was added to each sensor solution to make a total volume of 200 μL in acetic acid, separately. After stirring the solution for a few seconds, the absorption spectra were recorded at absorption maximum wavelength. The plots were drawn by plotting A_o_/(A_o_ − A) vs. 1/[Fe^3+^], where A_o_ equaled to absorption intensity of SQ without Fe^3+^, A was corresponded to the absorption intensity of SQ with different concentration of Fe^3+^. The Job’s plot procedures for SQ towards Hg^2+^ were similar as above. HgCl_2_ water solution and 4 mM SDS buffer were used instead.

### 2.4. Competition Tests

2 μL of NaCl, KCl, LiBr, AgNO_3_, ZnCl_2_, HgCl_2_, CdSO_4_, FeCl_2_, CoCl_2_, CaCl_2_ salt solutions (stock: 10 mM) was extracted individually and mixed with 2 μL Fe^3+^ (stock: 2 mM), 2 μL SQ (10 mM) and filled up with acetic acid to total volume of 200 μL. After stirring the solutions for a few seconds, UV-Vis spectra were recorded at room temperature. For the case of Hg^2+^ detection, HgCl_2_ solution was used instead of FeCl_3_. 

## 3. Results

### 3.1. Spectral Properties of SQ

The synthesis of sensor SQ was followed as reported literature [48] and the characterization for the compound was summarized in Figures S1–S3. The product was distinct from common squaraine as two carbonyl groups (C=O) on squaric core were adjacent to form 1,2-regioisomer instead of 1,3-regioisomer. It has two cross-conjugated-electron systems from hetero aromatic donor to the squaric core as donor acceptor. There are potential binding sites including two carbonyl groups (C=O) on squaric core and sulfur atom on the substituted 3-butyl-2-methylbenzo[d]thiazol-3-ium iodide which could provide extra electron pairs to the metal ions. There are many literatures that reported 1,3-regioisomer can be applied in the recognition of several metal ions [49,50,51,52]. We believe that besides 1,3-regioisomer, 1,2-regioisomer may also function as a metal ion detector in some circumstances.

**Figure 1:**
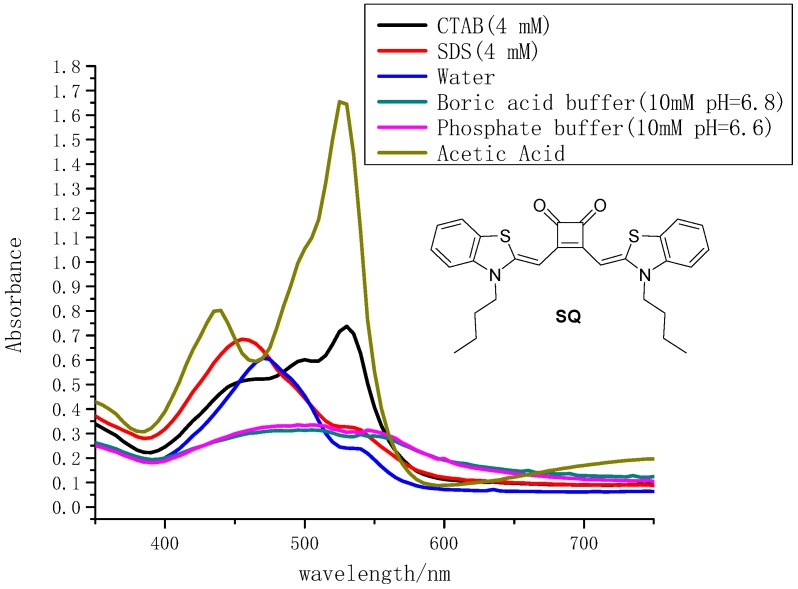
The absorption spectra of SQ (100 μM) in different solutions.

An important characteristic of squaraines is their tendency to form different patterns of aggregations, resulting in a dramatic color modulation [53,54]. We carried out the dilution experiments in some selected solvents (pure acetic acid and 4 mM SDS solution) by UV-Vis measurements to see if there were aggregations for SQ and the results were shown in supporting information (Figure S4a,b). It has shown that the absorption intensities decreased both in pure acetic acid and 4 mM SDS solution with the decrement of the SQ concentrations (10^−4^–10^−6^ M). The linear range of concentration of SQ in two different solvents was 1 μM to 10 μM by using the Beer–Lambert law. No absorption wavelength of SQ was shifted which suggest SQ was sensitive to the solvent environment but no aggregation can be concluded from the current data. The molar absorption coefficient of SQ in the linear range was not changed. A series of other solvents was applied in the UV-Vis measurements. Two microliters was extracted from 10 mM SQ stock solution and diluted with 198 μL corresponding solvent to get the total volume of 200 μL. The final concentration of SQ was 100 μM (100 times dilution). The absorption data for SQ in different solutions were summarized in Figure 1. SQ exhibited a maximum absorption wavelength at 475 nm in pure distilled water. While in boric acid buffer (10 mM, pH = 6.8) and phosphate buffer (10 mM, pH = 6.6), it gave a broad flat band in absorption range of 400 nm to 600 nm. Interestingly, it was found that in pure acetic acid, SQ showed two maximum absorption peaks individually at 430 nm and 525 nm. The addition of the cationic surfactant hexadecyl trimethyl ammonium bromide (CTAB) gave a relative broad absorption peak with maximum wavelength of 530 nm. SQ in anionic surfactant sodium dodecyl sulfonate (SDS) solution exhibited slight blue shift to 455 nm in the absorption spectra compared to that in the distilled water. 

### 3.2. Colorimetric Sensing for Fe^3+^ and Hg^2+^

**Figure 2:**
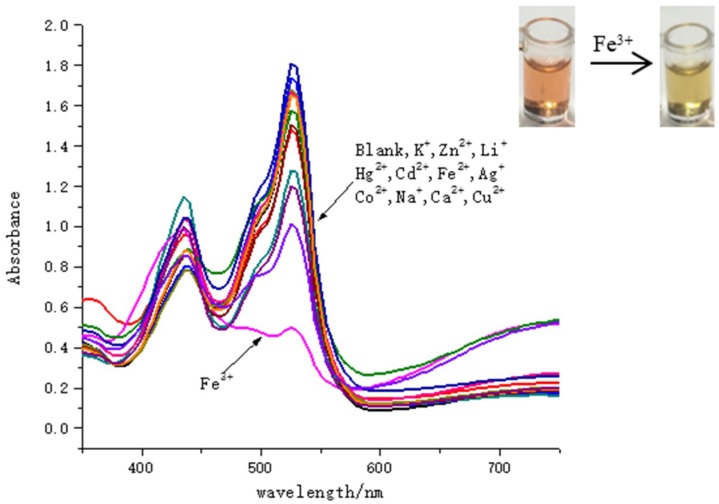
Absorption spectra for 3,4-bis((Z)-(3-butylbenzo[d]thiazol-2(3H)-ylidene)methyl) cyclobut-3-ene-1,2-dione (SQ) solution (100 μM) with/without addition of different metal ions (100 μM) in AcOH.

**Figure 3:**
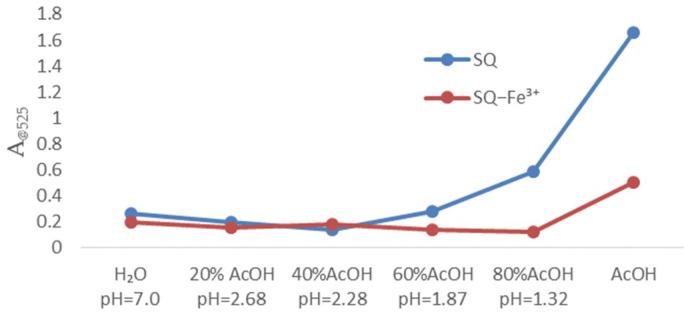
H_2_O effects in acetic acid for the SQ (100 μM) absorption changes with or without addition of Fe^3+^ (100 μM).

To evaluate the sensing properties of SQ (final concentration: 100 μM) towards metal ions, the UV-Vis spectral changes were investigated with addition of various metal ions including Na^+^, K^+^, Li^+^, Ag^+^, Zn^2+^, Hg^2+^, Cd^2+^, Fe^2+^, Co^2+^, Ca^2+^, Fe^3+^ (final concentration: 100 μM) in pure acetic acid. As shown in Figure 2, there were no significant spectral changes in the presence of most metal ions, whereas Fe^3+^ caused the distinct spectral changes with absorption intensity decreased in 525 nm. And Fe^3+^ pronounced color changes from orange to cream yellow by direct visualization. The water effect on detection of Fe^3+^ for SQ in AcOH-H_2_O solutions was further evaluated (Figure 3). There were gradually decreased absorption bands at 525 nm of SQ with increasing portion of water in acetic acid. The pH effect on the selectivity of SQ for Fe^3+^ has been observed as well. With increasing amount of AcOH in solution, the pH decreased gradually but the absorption intensity of SQ itself dramatically enhanced. In addition, only a distinct difference in absorption bands was observed in pure acetic acid for SQ with or without addition of Fe^3+^. We have also monitored the absorption changes of SQ in pure acetic acid and after 24 h, there were no significant changes in view of UV-Vis spectral (Figure S5a). In addition, ^1^HMR spectrum of SQ in CD_2_Cl_2_ after 24 h (Figure S5b) shown with no decomposition preliminarily revealed its stability in this condition. All these results indicated that SQ can be a good probe for detecting Fe^3+^.

**Figure 4:**
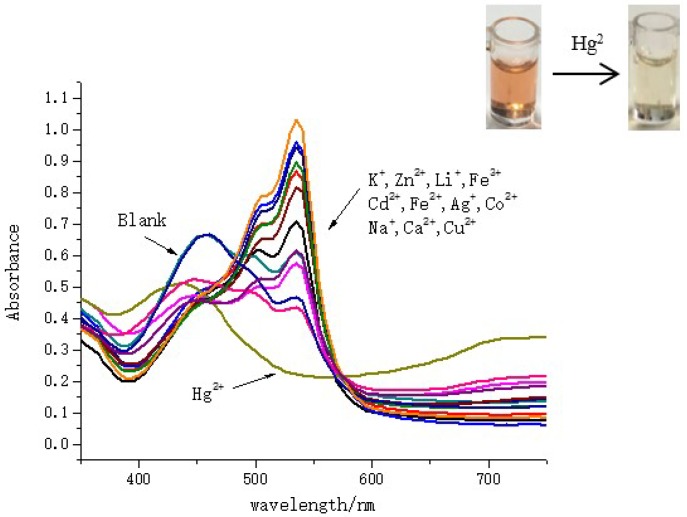
Absorption spectra for SQ solution (100 μM) with/without addition of different metal ions (100 μM) in sodium dodecyl sulfonate (SDS) (4 mM) solution.

Another interesting phenomenon was discovered in the detection of Hg^2+^ in SDS surfactant solutions. As shown in Figure 4, when various metal ions were added into SQ solutions in presence of 4 mM SDS, the single absorption band of SQ at 455 nm (maximum absorption wavelength without metal ions) was red shift to 535 nm except for Hg^2+^. Upon addition of Hg^2+^, the absorption band at 535 nm decreased gradually, while the absorption in the range of 650 nm to 750 nm increased with one clear isosbestic point, which indicating the binding between SQ and Hg^2+^ afforded only one species. This new peak might be ascribed to a metal-to-ligand charge transfer [55,56]. 

**Figure 5:**
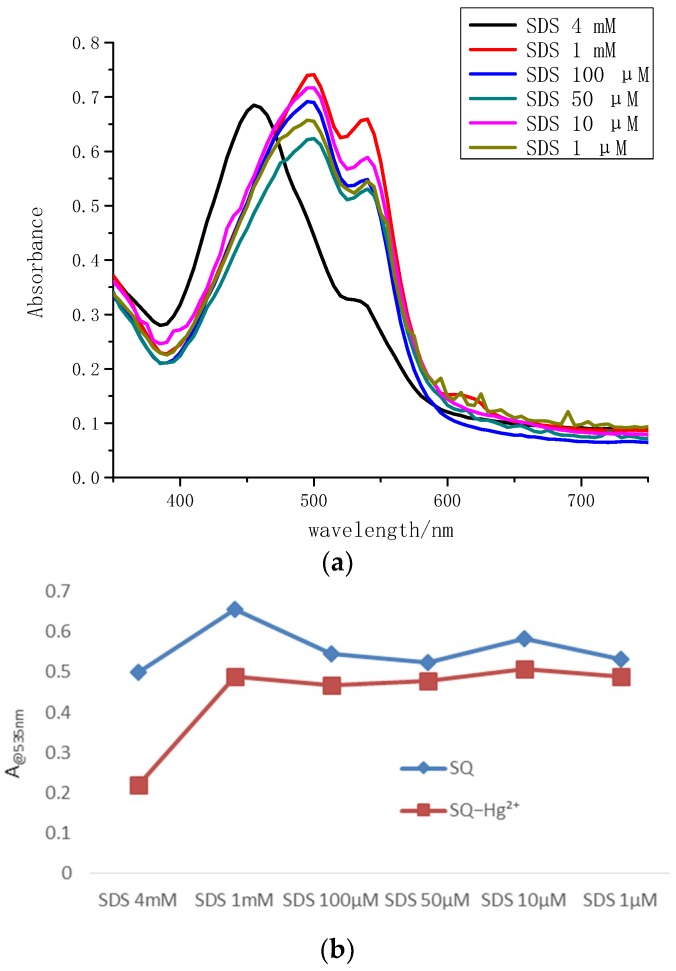
(**a**) The absorption spectra of SQ (100 μM) in different concentration of SDS (1 μM–4 mM) without addition of metal ions. (**b**) The relative changes of SQ (100 μM) absorption intensity in 4 mM SDS micellar solution at 535 nm with or without addition of Hg^2+^ (100 μM).

To evaluate the SDS effect on the Hg^2+^ detection, wide concentrations (1 μM–4 mM) of SDS solutions were adopted in Figure 5a. It was shown that when SDS concentration reached at 4 mM which was higher than critical micelle concentration (CMC) of SDS, the maximum absorption wavelength of SQ without metal ions exhibited a blue shift to 455 nm. It was found that the relative changes of SQ absorption intensity in 4 mM SDS micellar solution at 535 nm discriminate the most compared to that in other concentrations in the case of Hg^2+^ detection (Figure 5b). The solution of SQ in 4 mM SDS displayed an orange color, and the addition of Hg^2+^ caused an instant vivid color change from orange to grain yellow. The difference in color response allowed SQ to easily distinguish between Fe^3+^ and Hg^2+^ in aqueous solution.

**Figure 6:**
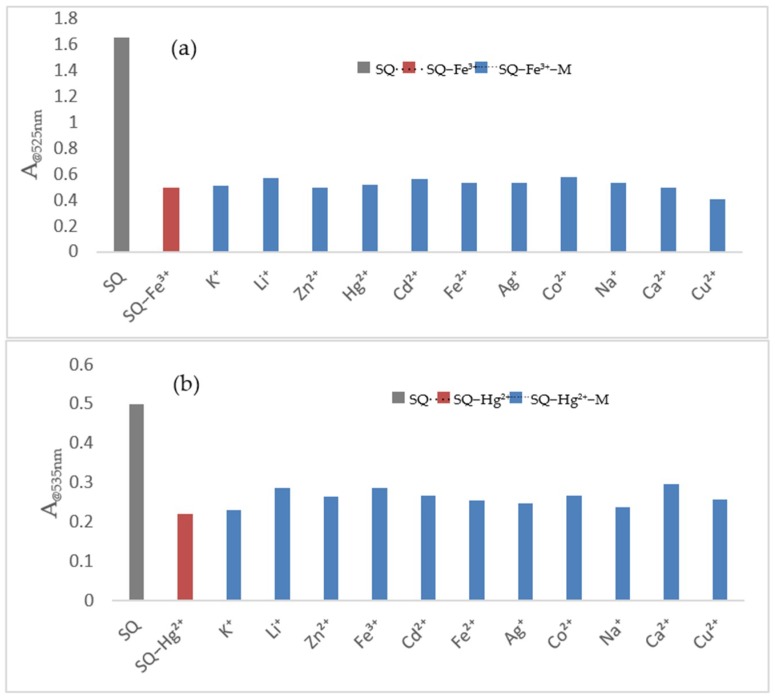
(**a**) Competition experiments of Fe^3+^ with other metal ions. Gray bar: absorption intensity of SQ (100 μM) in AcOH solution. Red bar: absorption intensity of SQ (100 μM) with Fe^3+^ (20 μM) in AcOH solution. Blue bar: absorption intensity of SQ (100 μM) with the addition of the respective competing cations (100 μM) and the Fe^3+^ (20 μM) in AcOH solution. (**b**) Competition experiments of Hg^2+^ with other metal ions. Gray bar: absorption intensity of SQ (100 μM) in SDS (4 mM) solution. Blue bar: absorption intensity of SQ (100 μM) with the addition of the respective competing cations (100 μM) in SDS (4 mM) solution. Red bar: absorption intensity of SQ (100 μM) with the Hg^2+^ (20 μM) in SDS (4 mM) solution. Blue bar: absorption intensity of SQ (100 μM) with the addition of the respective competing cations (100 μM) and the Hg^2+^ (20 μM) in SDS (4 mM) solution.

To validate the selectivity for Fe^3+^ and Hg^2+^, respectively, the competition experiments have also been performed. Other different ions as interferences were added to the mixture of SQ–Fe^3+^ or SQ–Hg^2+^ solution. The final concentration of other cations (100 μM) was 5 times more than that of Fe^3+^ (20 μM) and Hg^2+^ (20 μM) in the SQ solution. As shown in Figure 6a,b, there were no significant influences on Fe^3+^ and Hg^2+^ detections even extra amounts of other ions were added into the SQ–Fe^3+^ and SQ–Hg^2+^ system. All these findings suggest SQ exhibit high selectivity for Fe^3+^ and Hg^2+^ compared to other metal ions and it could be used as a colorimetric chemosensor for dual analytes analysis.

For the purpose of exploring the relationship between absorption intensity and response time, a dynamic study of SQ in the detection of Fe^3+^ and Hg^2+^ was carried out. After direct addition of Fe^3+^, the cream yellow color of SQ–Fe^3+^ was faded instantly and to total colorless after 10 h and for SQ–Hg^2+^, it took a longer time to get a total colorless solution (Figure S6a,b).

The reversibility and regeneration are essential for materials in practical applications. The reversibility of the recognition process of sensor SQ was performed by a reversible binding experiment. Ethylenediaminetetraacetic acid (EDTA) as a strong chelator was added into both SQ–Fe^3+^ and SQ–Hg^2+^ complex systems. Addition of 1 equivalent EDTA to SQ–Fe^3+^ system resulted in an increasing of absorption signal at 525 nm, indicating the regeneration of free SQ (Figure S7). With the alternate addition of constant concentrations of Fe^3+^ to SQ solution, the instant color change was observed again which exhibited good stability with a little signal decay for several cycles (Figure S8). For Hg^2+^, while after addition of 1 equivalent EDTA to SQ–Hg^2+^ system, a distinct absorption band with observed and the alternate addition of constant concentrations of Hg^2+^ has shown no dominant absorption changes (Figures S9 and S10). These clear findings of colorimetric ON/OFF behavior of the sensing system suggested that SQ serve as a good reversible sensor for Fe^3+^ detection.

### 3.3. Binding Constant (Ka) and Limit of Detection (LOD) for Fe^3+^ and Hg^2+^

**Figure 7:**
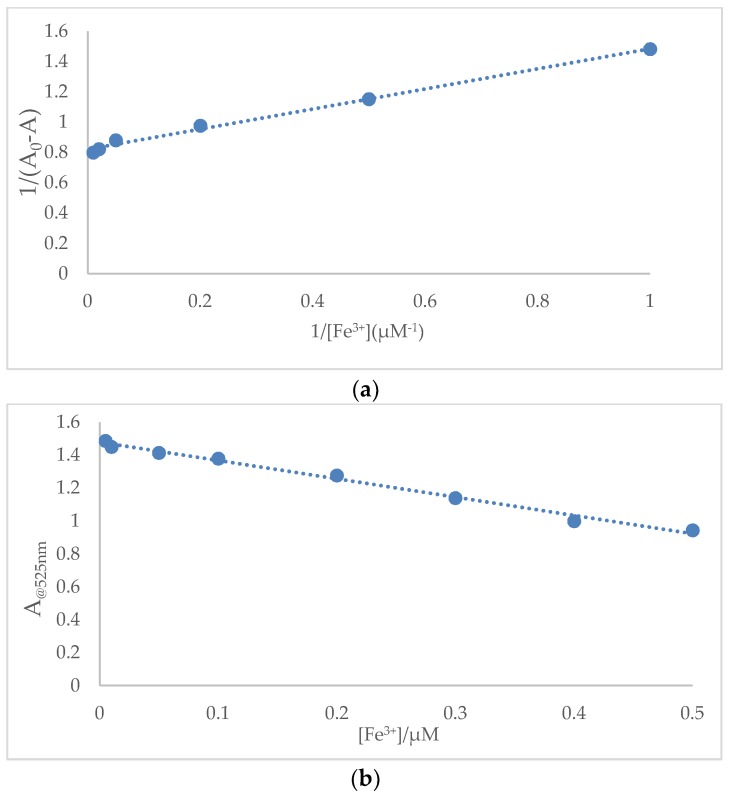
(**a**) Benesi-Hildebrand plot analysis of the absorption changes for the complexation between SQ and Fe^3+^, R^2^ = 0.9936. (**b**) Plot of absorption intensity change of SQ (100 μM) against concentrations of Fe^3+^ from 0.01 μM to 0.5 μM. R^2^ = 0.9907.

**Figure 8:**
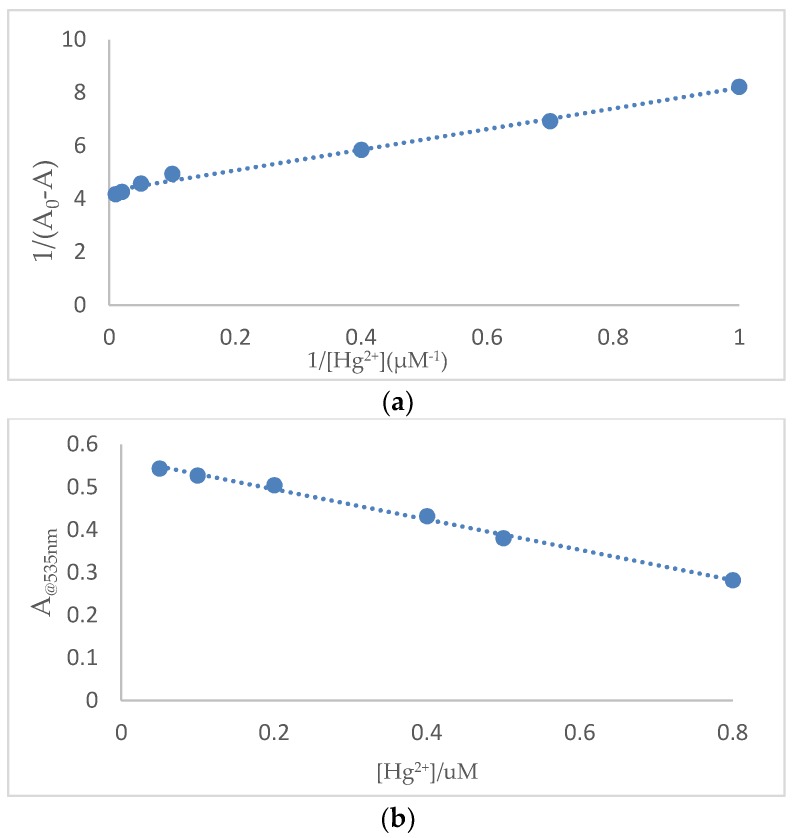
(**a**) Benesi-Hildebrand plot analysis of the absorption changes for the complexation between SQ and Hg^2+^, R^2^ = 0.9907. (**b**) Plot of absorption intensity change of SQ (100 μM) against concentrations of Hg^2+^ from 0.05 μM to 0.8 μM. R^2^ = 0.9948.

To get a further insight into the colorimetric sensing properties of SQ, a quantitative investigation of the binding affinity of sensor SQ with Fe^3+^ and Hg^2+^ was studied by titration. The UV-Vis absorption spectra of 100 μM SQ in acetic acid and SDS solution were recorded during the titration of various concentrations of Fe^3+^ (1 nM–100 μM) and Hg^2+^ (1 nM–100 μM), respectively. The binding constant (Ka) was estimated using a Benesi-Hilderbrand plot, which was calculated by absorption changes of consequent titration (A_0_/A_0_ − A) against 1/[M]. The magnitude of Ka was calculated from the intercept and slope of the straight line. The estimated value was about 1.24 × 10^6^ M^−1^ for SQ–Fe^3+^ complex and 1.1 × 10^6^ M^−1^ for SQ–Hg^2+^ (Figure 7a and Figure 8a). 

From the absorption titration, the detection limit for Fe^3+^ and Hg^2+^ on the basis of 3σ/k was therefore calculated and determined to be 0.538 μM (lower than the U. S. Environmental Protection Agency guideline for drinking water, 5.37 μM) and 1.689 μM (Figure 7b and Figure 8b) for Hg^2+^ which were superior than many recent reported Fe^3+^ and Hg^2+^ related sensors (Table 1). The limit of detection for Hg^2+^ can be further improved by modifying the structure through introducing more functional groups to increase the solubility of SQ and hydrogen interaction to the metal ions.

### 3.4. Complexation Mechanism of SQ–Fe^3+^ and SQ–Hg^2+^

**Figure 9:**
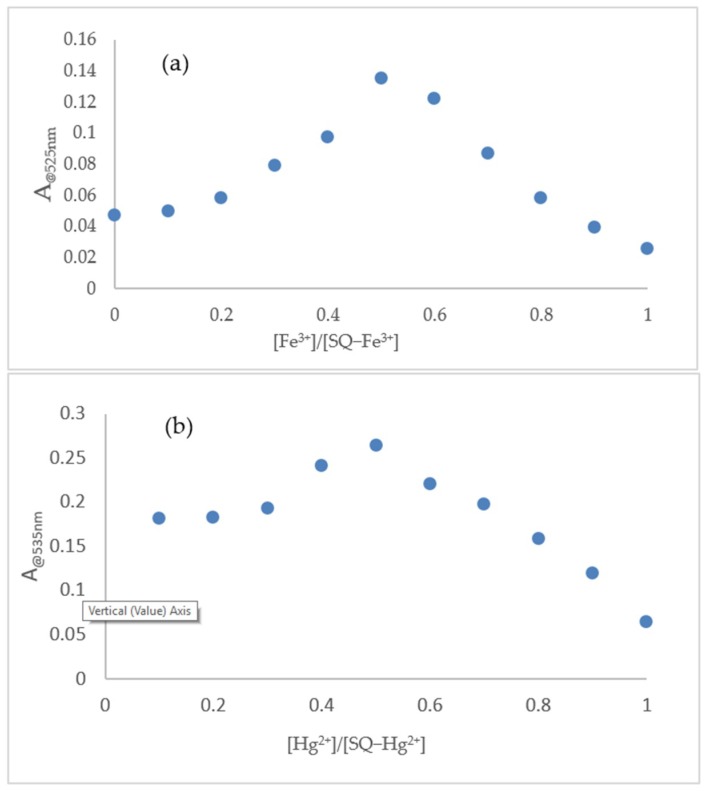
(**a**) Job’s plot for the complexation of SQ with Fe^3+^ in AcOH solution; (**b**) Job’s plot for the complexation of SQ with Hg^2+^ in SDS (4 mM).

Job’s plot measurement was carried out to determine the complexation mode between SQ and Fe^3+^. A maximum value of the absorption intensity at 525 nm was observed when the mole fraction of Fe^3+^ reached 0.5. A signature of 1:1 stoichiometry between SQ and Fe^3+^ was determined. In addition, a 1:1 stoichiometry between SQ and Hg^2+^ was also determined following the same process. (Figure 9a,b). The Electron Spray Ionization-Mass data of SQ–Fe^3+^ and SQ–Hg^2+^ complexes were included in Figures S11 and S12. 

**Figure 10:**
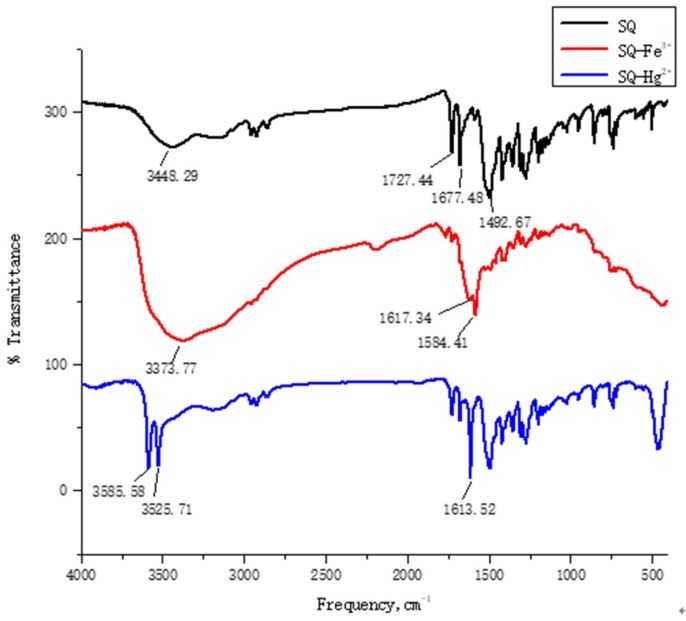
The IR spectrum of SQ (Black line), SQ–Fe^3+^ (Red line), and SQ–Hg^2+^ (Blue line) complex in the solid state.

**Scheme 1:**
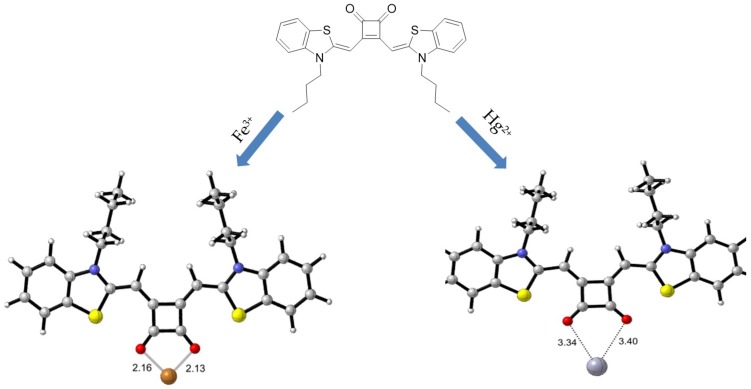
Proposed complexation mechanism of SQ–Fe^3+^ and SQ–Hg^2+^.

The involvement of binding sites of SQ in complexation was further confirmed through the IR analysis in the presence and absence of metal ions (Figure 10). The characteristic carbonyl stretching frequencies of SQ appeared at 1727.44 cm^−1^ and 1677.48 cm^−1^. However, in the SQ–Fe^3+^ complex, a new peak at 1617.34 cm^−1^ was observed instead of those at 1766 cm^−1^ and 1602 cm^−1^. At the same time, another peak appeared at 1584.41 cm^−1^ instead of 1492.07 cm^−1^, indicating the SQ binding with Fe^3+^ occurs at the two carbonyl groups of squarely moiety in SQ. The IR spectrum for SQ–Hg^2+^ complex was different to that of SQ–Fe^3+^ complex. The characteristic carbonyl stretching peak in SQ–Hg^2+^ complex at 1766 cm^−1^ and 1602 cm^−1^ were replaced by a strong new peak at 1613.52 cm^−1^. More importantly, two more new peaks at 3585.58 cm^−1^ and 3525.71 cm^−1^ shown up ascribed to proton vibration. This indicated that more electron pairs were involved in the binding with presence of Hg^2+^. Conceptually, in accordance with hard soft acid base (HSAB) principle [64], the complexations of SQ–Fe^3+^ and SQ–Hg^2+^ were undergone two different pathways, which have been proposed in Scheme 1. For SQ–Fe^3+^ complex, Fe^3+^ was proposed to bind two carbonyl groups in squaric moiety with 1:1 stoichiometry, while for SQ–Hg^2+^ complex, it carried out in a different way.

**Figure 11:**
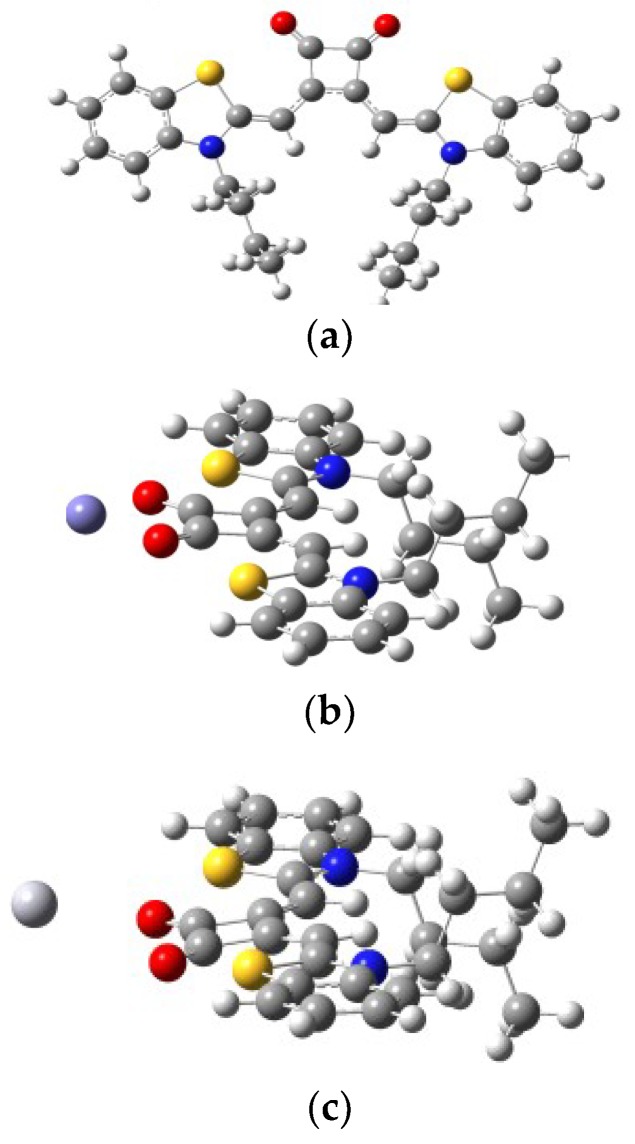
The geometry-optimized structures of (**a**) SQ (yellow, red and blue atoms representing as sulfur, oxygen, nitrogen atoms, respectively); (**b**) SQ–Fe^3+^ complex (purple atom representing as Fe^3+^); (**c**) SQ–Hg^2+^ complex (individual atom at left representing as Hg^2+^ atom) at the SMD (H_2_O)-TD-PBE0/TZVP+LANL2DZ level.

Theoretical calculations have been explored as well to understand the nature of the binding of SQ–Fe^3+^ and SQ–Hg^2+^ complexes. All calculations were carried out with the Gaussian 09 package [65]. The density functional theory (DFT) hybrid model with the B3LYP was used for the gas-phase geometry optimization, Lanl2dz basis set with effective core potential (ECP) for Fe and Hg, and the 6-31G(d) basis set was used for all remaining atoms. Based on the calculations, the favorable binding modes between Fe^3+^ and its Hg^2+^ complexes were depicted in Figure 11a–c. According to the calculations, the geometry optimized structure of SQ–Fe^3+^ complex (Figure 11b) was illustrated the same as our proposed. It showed lower energy when Fe^3+^ was bind two carbonyl groups in squarely moiety. However, the simulated spectra are in good agreement with the proposed complexation mechanism for only SQ–Fe^3+^ complexes. For SQ–Hg^2+^ complex, the Hg atom was far away from the SQ. 

### 3.5. Preliminary Analytical Application

The SQ has been validated for practical applications in the determination of Fe^3+^ and Hg^2+^ in industry waste water (Table 2). Control experiments showed no significant effect on the colorimetric change of the sensor, the samples were used for the spike and recovery test after treating with acetic acid and 4 mM SDS solution, respectively. It was revealed that SQ has shown a good recovery at different concentrations (Figure S13a,b). These results preliminarily demonstrated that SQ could be potential to be used in selectively and sensitively determine Fe^3+^ and Hg^2+^ in real water samples.

## 4. Conclusions

In conclusion, a simple 1,2-squaraine SQ has been developed to performance dual colorimetric sensing for Fe^3+^ and Hg^2+^ ions. An instant color change for selective Fe^3+^ detection in pure acetic acid was observed with a detection limit of 0.538 μM, while Hg^2+^ can be detected selectively in 4 mM SDS solution by instant colorimetric response with a detection limit of 1.689 μM. The Job’s plot supported the 1:1 biding mode for both SQ–Fe^3+^ and SQ–Hg^2+^ complexes. IR analysis and DFT calculations demonstrated the SQ–Fe^3+^ and SQ–Hg^2+^ complexes undergo a different complex mechanism. Our findings provide a simple material and a facile, low cost colorimetric method for dual analytes analysis and have shown preliminary analytical applications in industrial water samples. 

## Figures and Tables

**Table 1:** Comparison of SQ with recently reported chemosensors.

Sensor	Target	Response Type	LOD (μM)	Reaction Media	Reversibility	Reference
Dansyl based derivative	Fe^3+^	Fluorescence	0.62	C_2_H_5_OH-H_2_O (1:1, *v*/*v*)	No	[57]
Rhodamine derivative	Fe^3+^	Fluorescence	0.74	CH_3_CN-H_2_O(1:1, *v*/*v*)	Yes	[58]
Sugar-functioned coumarin	Fe^3+^	Color	4.6	H_2_O	No	[59]
Julolidine derivative	Fe^3+^	Color	6.8	DMF	No	[57]
Squaraine -bis(rhodamine-B) derivative	Hg^2+^	Fluorescence	6.48	CH_3_CN	Yes	[60]
Coumarin-urea derivative	Hg^2+^	Fluorescence	0.45	CH_3_CN	Yes	[61]
Hetarylazo	Fe^3+^	Color	2.0	CH_3_CN	No	[62]
	Hg^2+^	Color	2.0	CH_3_CN	No	
Naphthalimide-rhodamine	Fe^3+^	Color	0.57	EtOH/PBS buffer (1:1)	No	[63]
	Hg^2+^	Fluorescence	2.72	EtOH/PBS buffer (1:1)	Yes	
Our work	Fe^3+^	Color	0.54	CH_3_COOH	Yes	/
	Hg^2+^	Color	1.69	SDS (4 mM)	No	

**Table 2:** Determination of ion in industrial waste water samples with SQ.

Sample		AAS (μM)	Added (mM)	Found (mM)	Recovery (%)
Industrial waste water I	Fe^3+^	2.01	1.12	1.14	102
	Hg^2+^	3.20	4.30	4.41	102
Industrial waste water II	Fe^3+^	4.03	2.23	2.19	98
	Hg^2+^	2.43	2.85	2.77	97
Industrial waste water III	Fe^3+^	6.05	4.41	4.38	99
	Hg^2+^	1.65	2.20	2.17	99

## Supplementary Materials

The following are available online at http://www.mdpi.com/1996-1944/11/10/1998/s1; Figure S1: ^1^H NMR spectrum for compound SQ; Figure S2: ^13^C NMR spectrum for compound SQ; Figure S3: high resolution mass spectrum for SQ; Figure S4: (a) absorption for different concentrations of SQ in pure acetic acid and (b) absorption for different concentrations of SQ in 4 mM SDS solution; Figure S5: (a) absorption of SQ in pure acetic acid at different time points; (b) ^1^HMR spectra of SQ in CD_2_Cl_2_ after 24 hours; Figure S6: (a) dynamic study on the absorption change of SQ–Fe^3+^ and (b) dynamic study on the absorption change of SQ–Hg^2+^, Figure S7: reversible study of SQ–Fe^3+^ toward addition of EDTA; Figure S8: reversible study of SQ–Hg^2+^ upon alternate addition of Fe^3+^; Figure S9: reversible study of SQ–Hg^2+^ complex toward addition of EDTA; Figure S10: reversible absorption changes of SQ upon alternate addition of Hg^2+^ and EDTA; Figure S11: high resolution mass spectrum for SQ–Fe^3+^, Figure S12: high resolution mass spectrum for SQ–Hg^2+^; Figure S13: (a) titration curve of SQ for Fe^3+^ (1–6 mM) and (b) titration curve of SQ for Hg^2+^ (1–6 mM).
