# Supplementary material for: Dual Sensing Performance of 1,2-Squaraine for the Colorimetric Detection of Fe3+ and Hg2+ Ions
Source: Materials (Basel). 2018 Oct 16;11(10):1998. doi: 10.3390/ma11101998 (PMC6212916; doi:10.3390/ma11101998)
Supplement: Supplementary file 1 [file materials-11-01998-s001.pdf]

Supplementary Materials: Dual Sensing Performance of 1,2-Squaraine for the Colorimetric Detection of Fe^{3+} and Hg^{2+} Ions

Xiaoqian Liu [1,*], Na Li [1], Min-Min Xu [2], Chunhui Jiang [3], Jianhao Wang [1], Guoqiang Song [1,*] and Yong Wang [2,*]

1. General Information

Reagents and Apparatus

Unless stated, all the reagents used were of analytical grade from commercial sources without further purification. ^{1}H NMR (400 MHz) and ^{13}C NMR (400 MHz) spectra were recorded on a Bruker AV-400 spectrometer (TMS as internal standard). Mass spectrometry analysis was performed on a Q exactive mass spectrometer (Thermo Fisher Scientific, Waltham, MA, USA). Absorption spectra were measured on M5 spectrometer.

Nuclear Magnetic Resonance Spectroscopy

Proton nuclear magnetic resonance (^{1}H NMR) and carbon nuclear magnetic resonance (^{13}C NMR) spectroscopy were performed on 400 MHz NMR spectrometers. Chemical shifts are reported as δ in units of parts per million (ppm) downfield from tetramethysilane (δ0.00), using the residual solvent signal as an internal standard: chloroform-d, $CDCl_3$, (^{1}H NMR, δ7.26, singlet; ^{13}C NMR, δ77.04, triplet). Multiplicities are given as: s (singlet), d (doublet), t (triplet), q (quartet), m (muliplets). The number of protons (n) for a given resonance is indicated by numbers of H.

UV-vis Titration Measurements

10 mM stock solution of SQ was prepared by dissolving the required amount in DMSO. Further dilutions were made to prepare 100 μM of TSQ by adding mixed solution. The different concentrations of metal ions were consequently were added in to make total volume of 200 μL. Absorption measurements were made in 96 well plates.

2. NMR Spectra for SQ

The synthetic procedures of sensor SQ were illustrated in Scheme 1, by a condensation reaction of 3-butyl-2-methylbenzo[d]thiazol-3-ium iodide (0.2 g, 0.6 mmol) and ethyl squarate (0.05 g, 0.3 mmol) mixture in 4 ml ethanol together with 0.06 g trimethylamine refluxing for 8 h [50]. After cooling to room temperature, the solvent was evaporated from the reaction and the resulting mixture was purified by column chromatography using DCM: MeOH = 30:1 as eluent to afford compound 3,4-bis((Z)-(3-butylbenzo[d]thiazol-2(3H)-ylidene)methyl) cyclobut-3-ene-1,2-dione (SQ). The yield was 60.5% and SQ was confirmed by NMR and ESI-MS [Figure S1–S3]. ^{1}H NMR (400 MHz, CD_2Cl_2) δ: 7.47–7.45(d, J = 8.0 Hz, 1H), 7.34–7.29(m, 1H), 7.12–7.04(m, 2H), 5.44(s, 1H), 1.84–1.77(m, 2H), 1.57–1.48(m, 2H), 1.06–1.02(m, 3H). ^{13}C NMR (400 MHz, CD_2Cl_2) δ: 177.55, 157.14, 141.45, 128.14, 126.98, 126.53, 122.78, 121.79, 110.40, 80.99, 53.98, 53.71, 53.44, 53.17, 52.90, 45.39, 28.54, 20.27, 13.58. HR-ESI-MS Calculated for: 488.1592; Found: 488.1589.

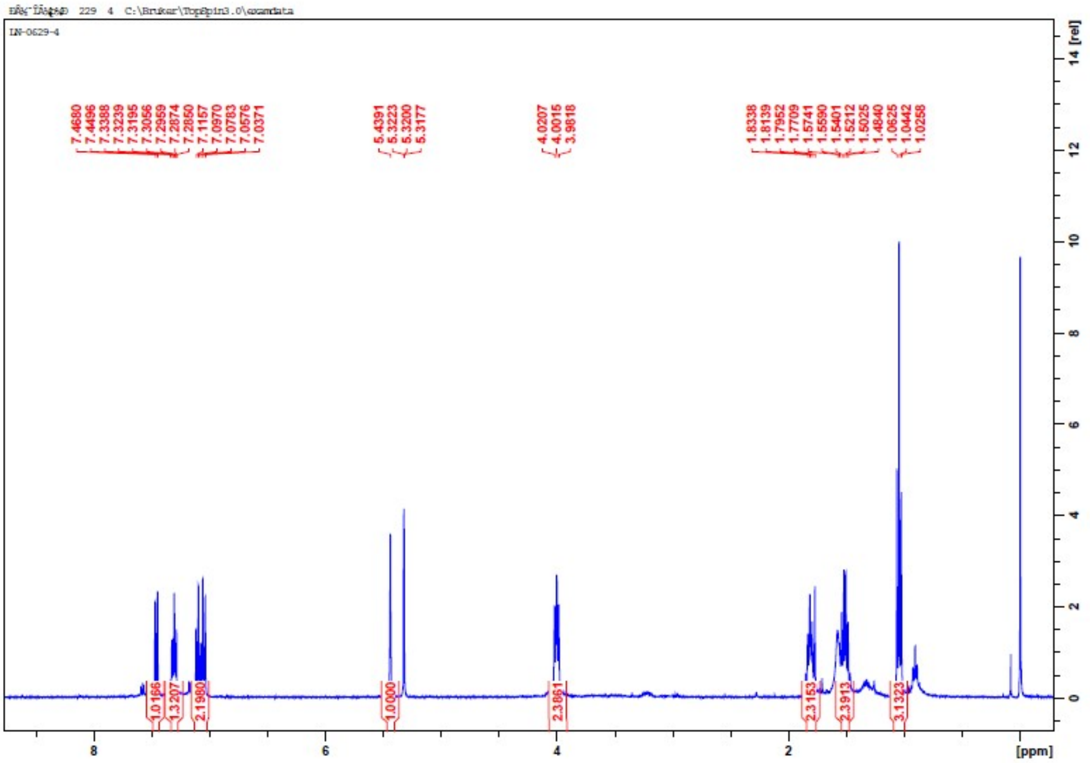

Figure S1. ^{1}H NMR spectrum for compound SQ.

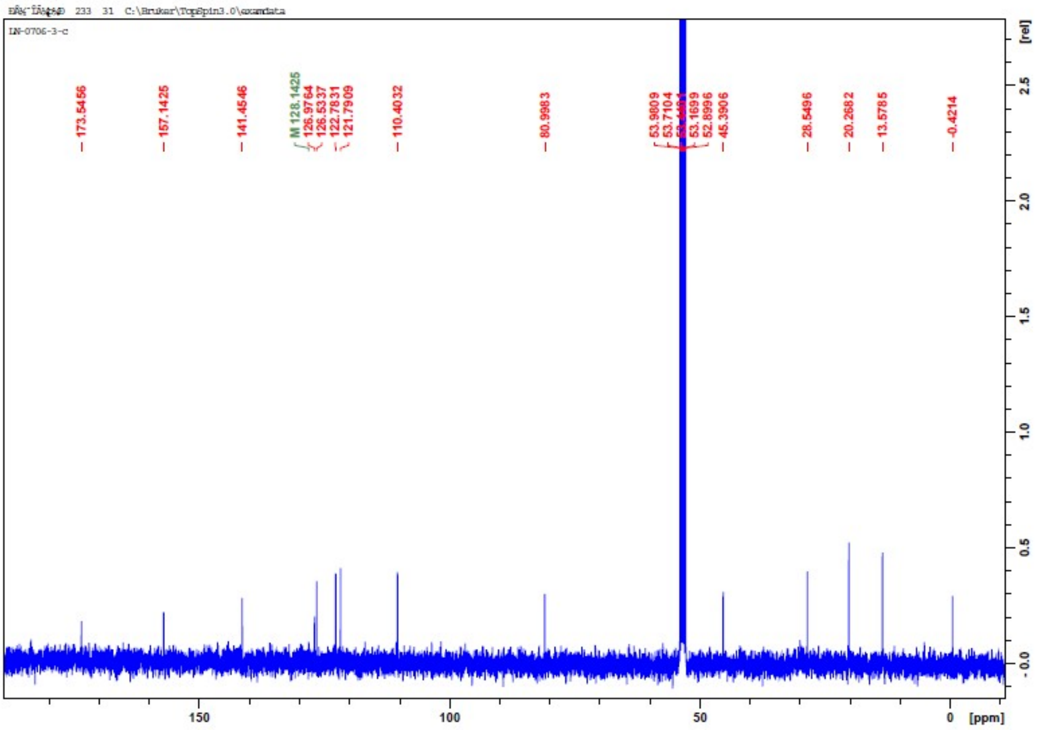

Figure S2. ^{13}**C NMR** spectrum for compound SQ.

3. Mass Spectrum of SQ

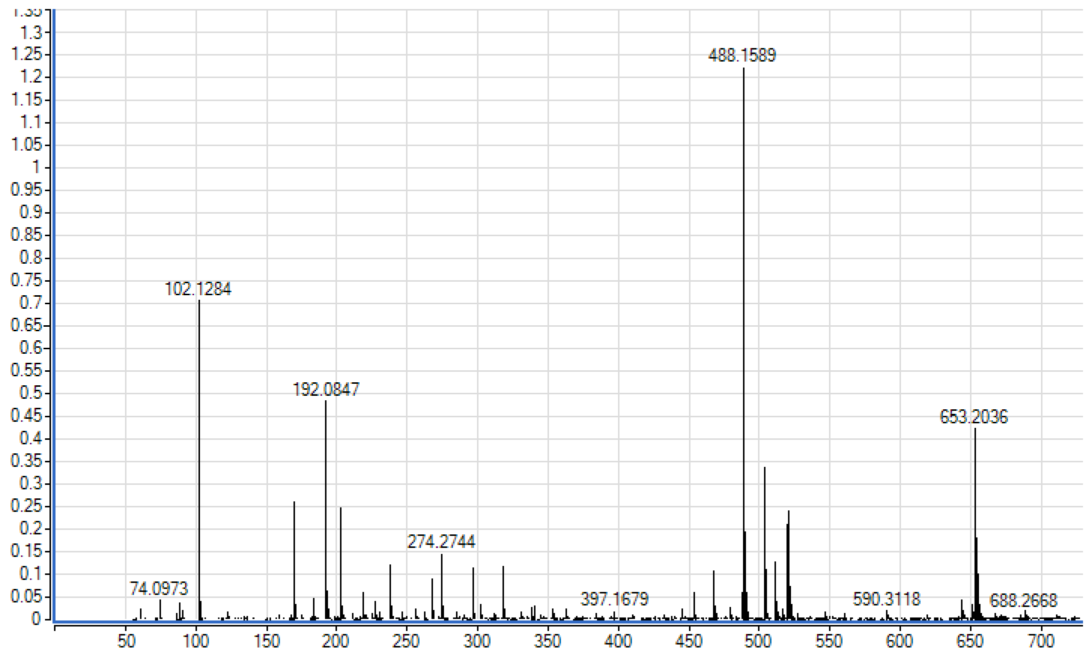

Figure S3. High resolution mass spectrum for SQ.

4. Absorption of SQ with Diluted Concentrations in Selected Solutions.

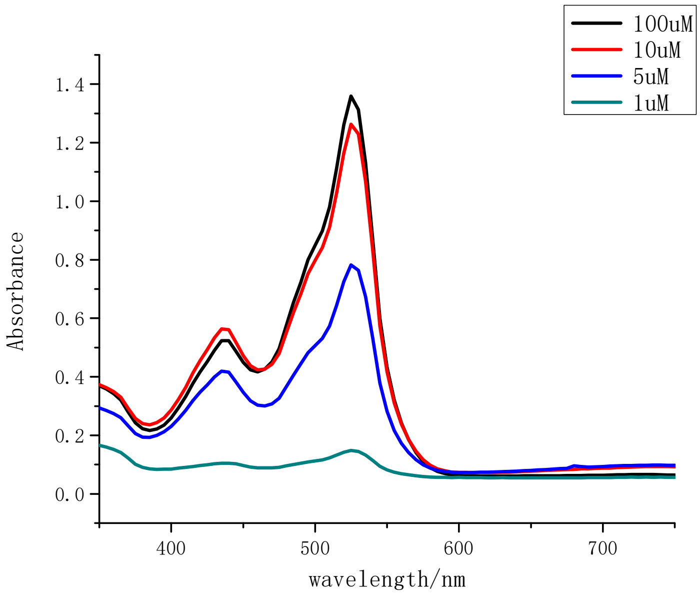

(**a**)

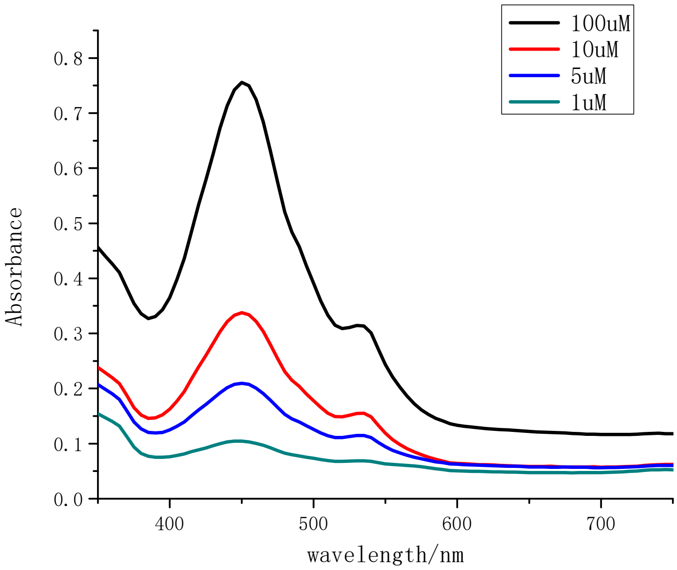

(**b**)

Figure S4. (**a**)Absorption for different concentrations of SQ in pure acetic acid, (**b**) Absorption for different concentrations of SQ in 4 mM SDS solution.

5. Absorption of SQ in Pure Acetic Acid at Different Time Points, [1]HMR of SQ in CD_2Cl_2 after 24 h

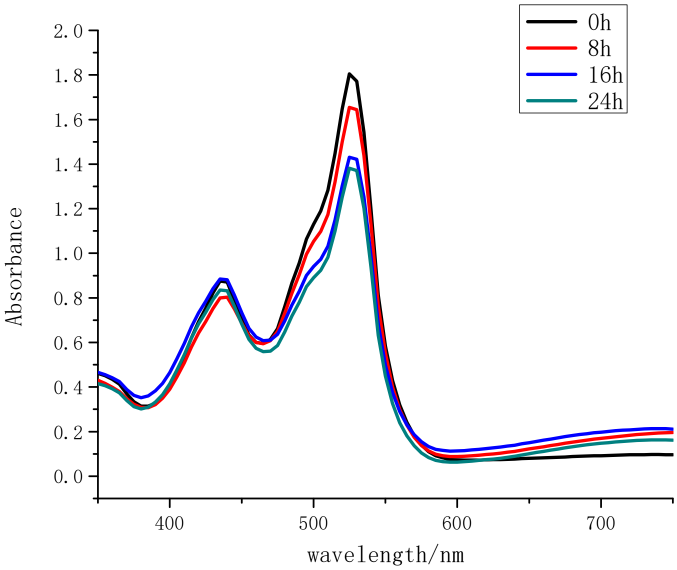

(**a**)

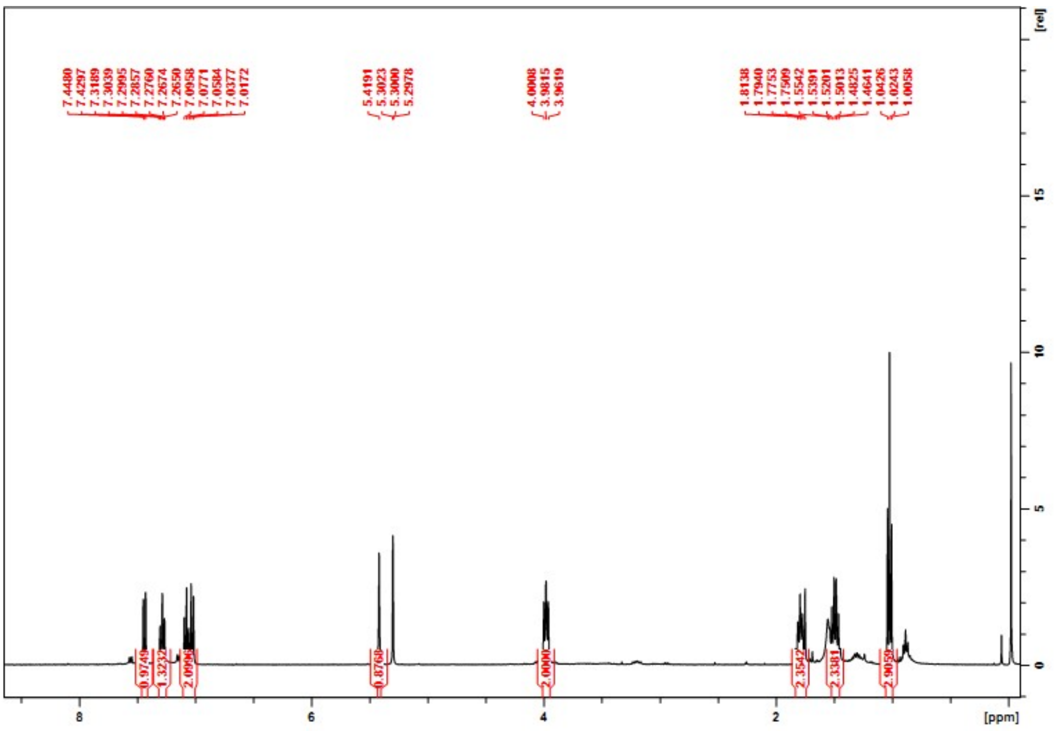

(b)

Figure S5. (**a**) Absorption of SQ in pure acetic acid at different time points, (**b**) [1]HMR spectra of SQ in CD_2Cl_2 after 24 hours.

7. Dynamic Study on the Absorption Change of SQ–Fe^{3+} and SQ–Hg^{2+}

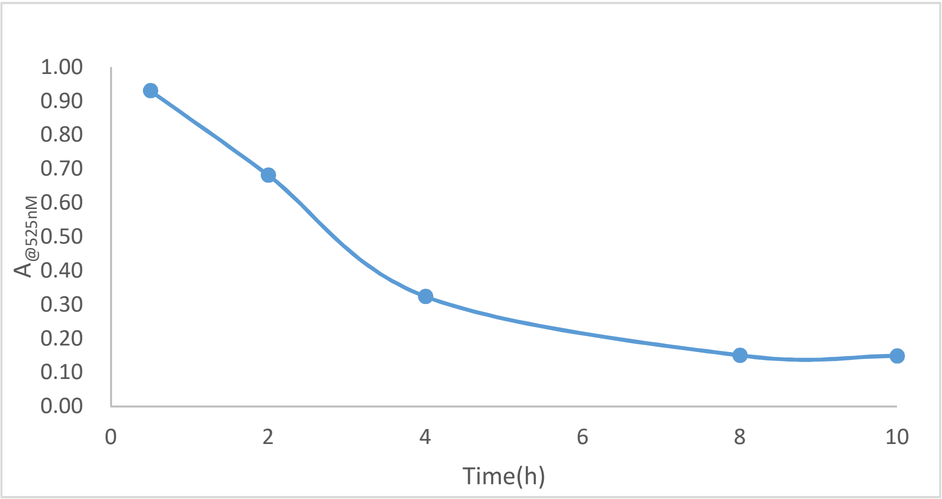

(a)

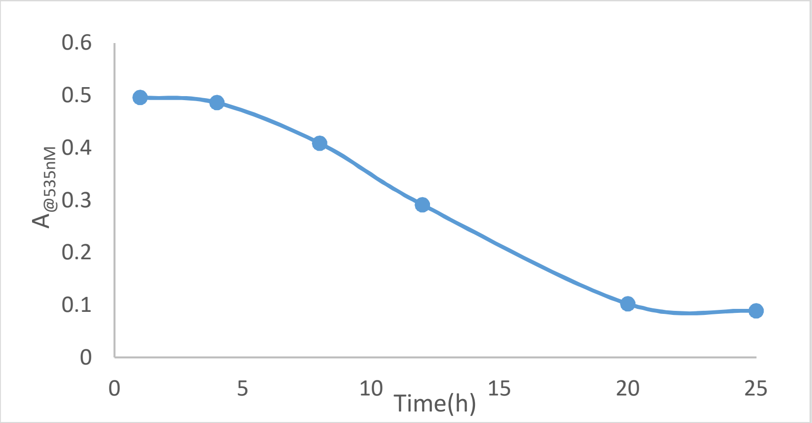

(**b**)

Figure S6. (**a**) Dynamic study on the absorption change by mixing SQ (100 μM) and Fe^{3+} (100 μM) in AcOH solution, (**b**) Dynamic study on the absorption change by mixing SQ (100 μM) and Hg^{2+} (100 μM) in AcOH solution.

8. Reversible Study of SQ–Fe^{3+} Complex

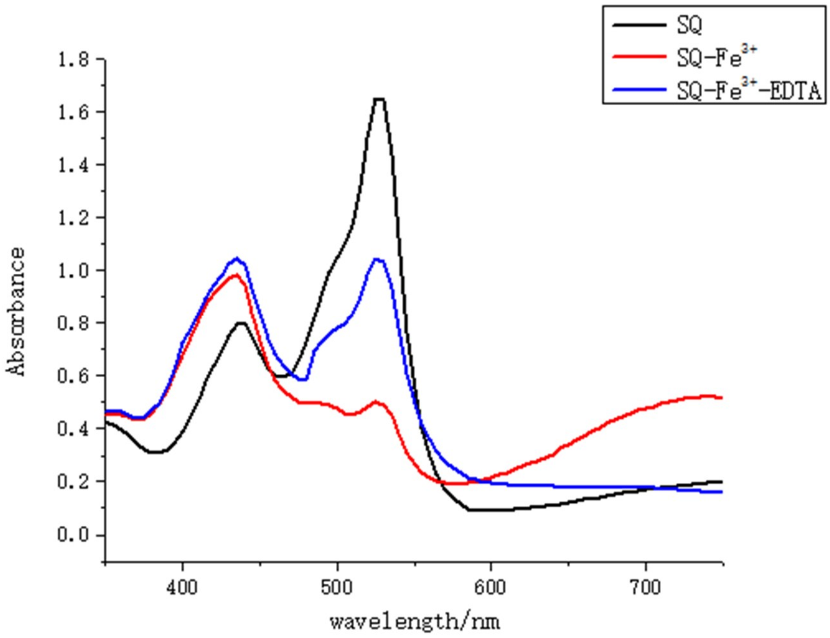

Figure S7. Reversible study of SQ–Fe^{3+} complex (100 μM) toward addition of EDTA (100 μM).

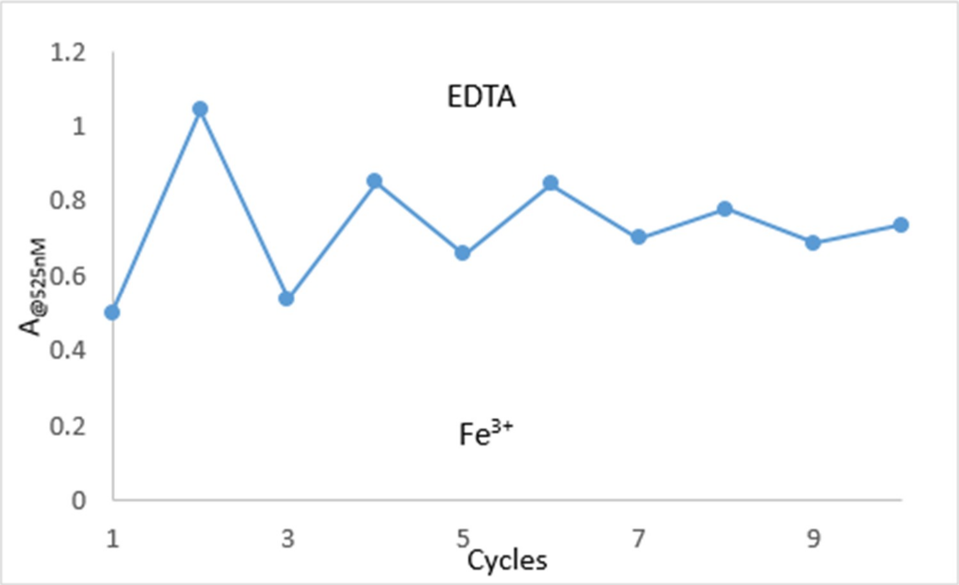

Figure S8. Reversible absorption changes of SQ upon alternate addition of Fe^{3+}.

9. Reversible Study of SQ–Hg^{2+} Complex

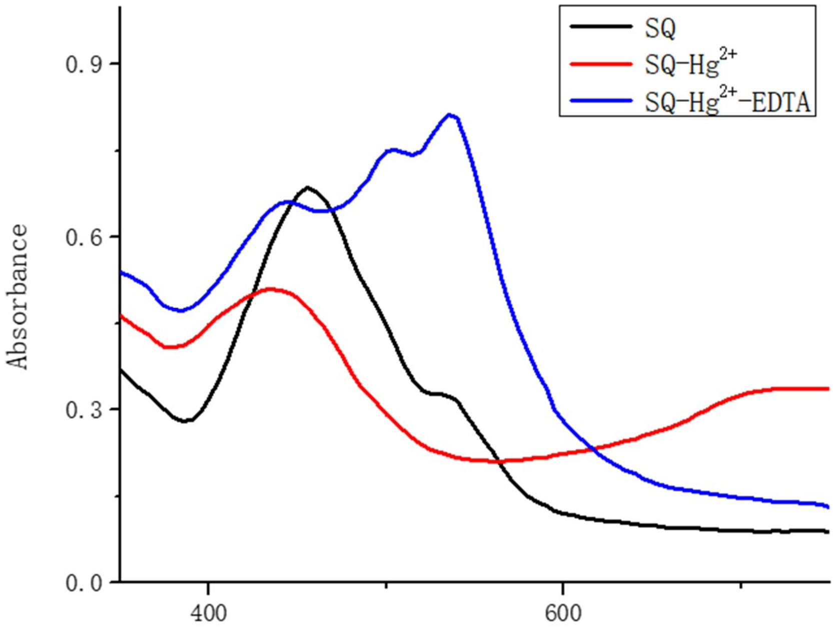

Figure S9. Reversible study of SQ–Hg^{2+} complex (100 μM) toward addition of EDTA (100 μM).

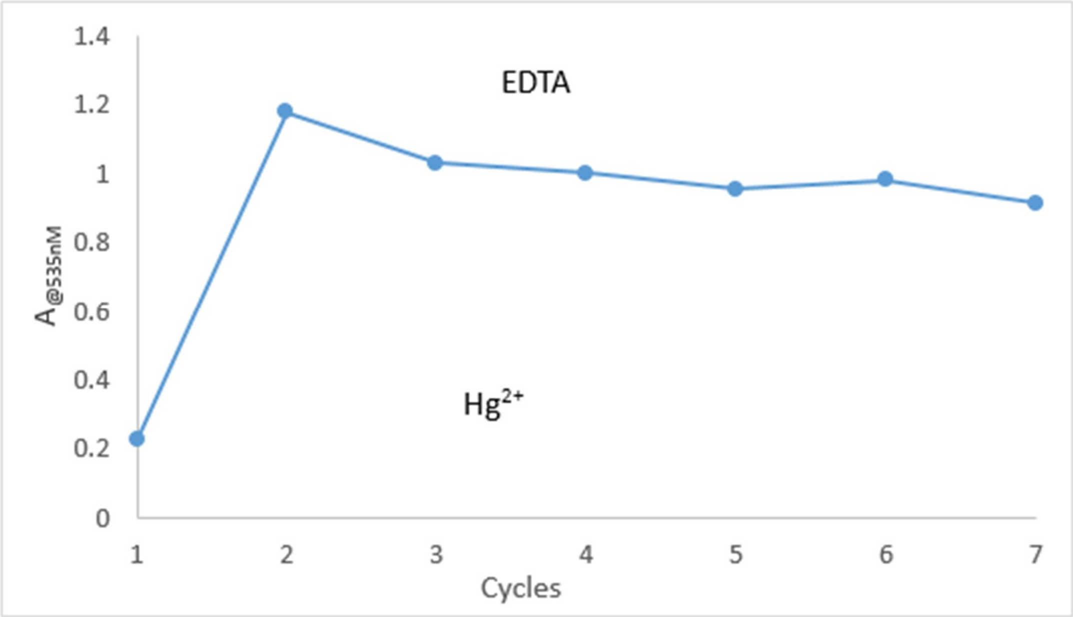

Figure S10. Reversible absorption changes of SQ upon alternate addition of Hg^{2+} and EDTA.

10. Mass Spectrum for SQ–Fe^{3+} and SQ–Hg^{2+}

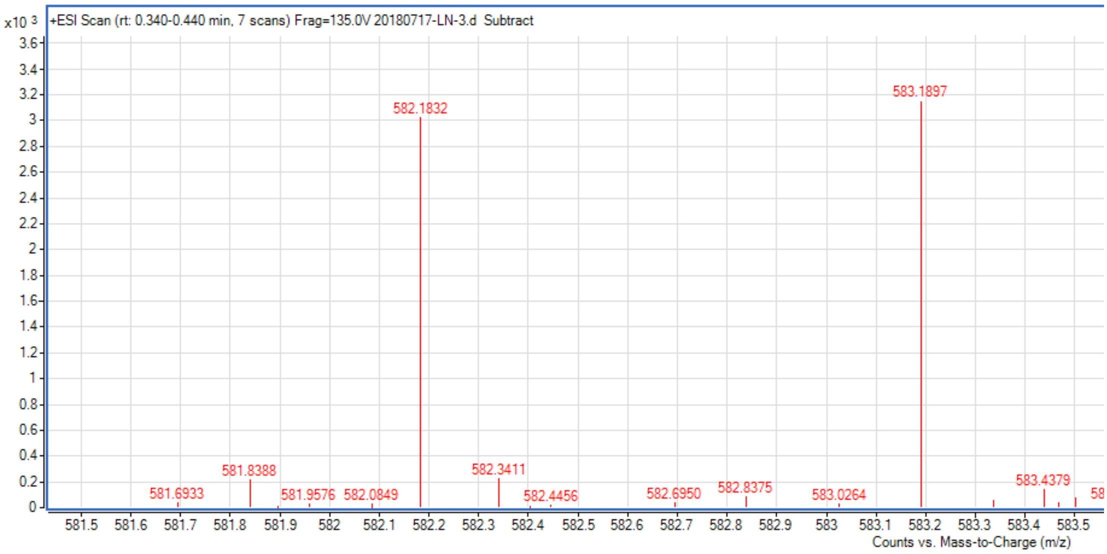

Figure S11. High resolution mass spectrum for SQ–Fe^{3+}.

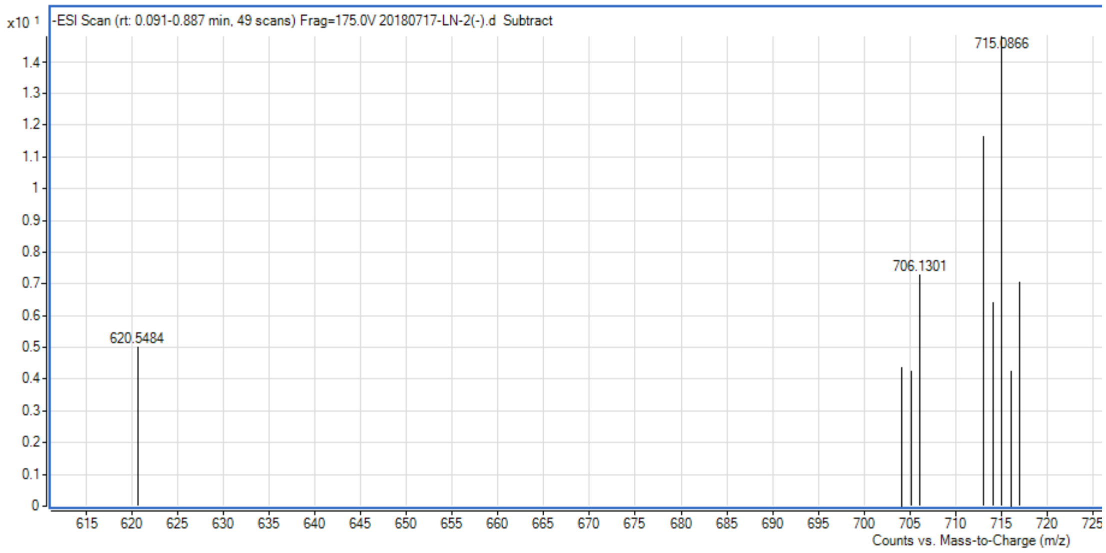

Figure S12. High resolution mass spectrum for SQ–Hg^{2+}.

11. Titration Curve of SQ for Fe^{3+} and Hg^{2+}

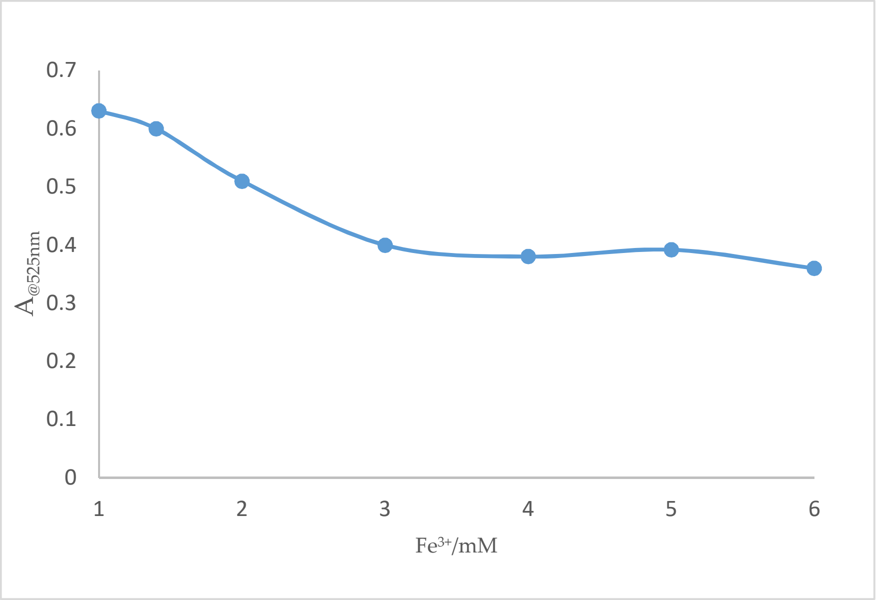

(a)

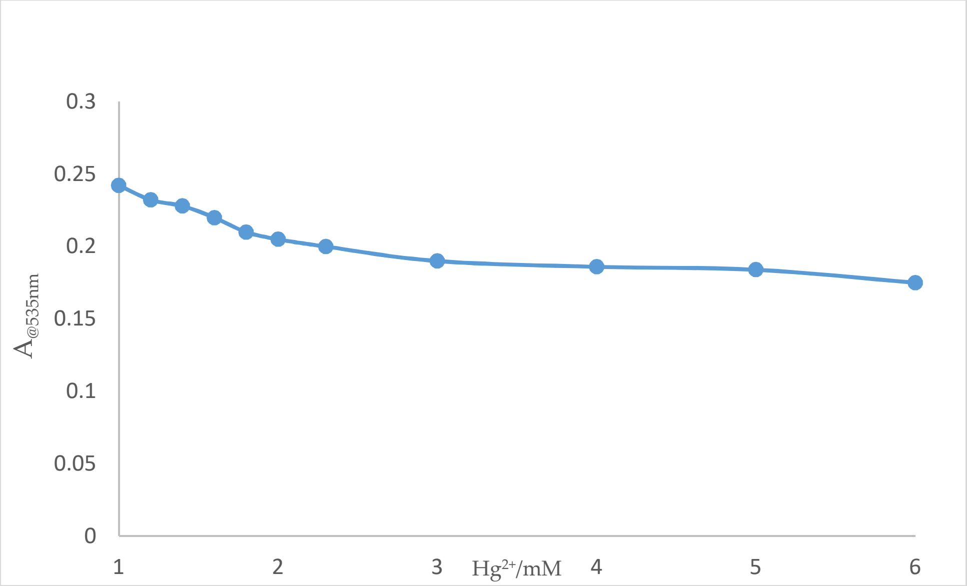

(**b**)

Figure 13. (**a**) Titration curve of SQ for Fe^{3+} (1–6 mM), (**b**) Titration curve of SQ for Hg^{2+} (1–6 mM).

© 2018 by the authors. Submitted for possible open access publication under the terms and conditions of the Creative Commons Attribution (CC-BY) license (http://creativecommons.org/licenses/by/4.0/).
